# Western Australian Marsupials Are Multiply Infected with Genetically Diverse Strains of *Toxoplasma gondii*

**DOI:** 10.1371/journal.pone.0045147

**Published:** 2012-09-24

**Authors:** Shuting Pan, R. C. Andrew Thompson, Michael E. Grigg, Natarajan Sundar, Andrew Smith, Alan J. Lymbery

**Affiliations:** 1 School of Veterinary and Biomedical Sciences, Murdoch University, Perth, Western Australia, Australia; 2 Molecular Parasitology Unit, Laboratory of Parasitic Diseases, National Institutes of Allergy and Infectious Diseases, Bethesda, Maryland, United States of America; 3 Fish Health Unit, School of Veterinary and Biomedical Sciences, Murdoch University, Perth, Western Australia, Australia; Obihiro University of Agriculture and Veterinary Medicine, United States of America

## Abstract

Five different organs from 16 asymptomatic free-ranging marsupial macropods (*Macropus rufus*, *M. fuliginosus*, and *M. robustus*) from inland Western Australia were tested for infection with *Toxoplasma gondii* by multi-locus PCR-DNA sequencing. All macropods were infected with *T. gondii*, and 13 had parasite DNA in at least 2 organs. In total, 45 distinct *T. gondii* genotypes were detected. Fourteen of the 16 macropods were multiply infected with genetically distinct *T. gondii* genotypes that often partitioned between different organs. The presence of multiple *T. gondii* infections in macropods suggests that native mammals have the potential to promote regular cycles of sexual reproduction in the definitive felid host in this environment.

## Introduction

*Toxoplasma gondii* is a ubiquitous intracellular protozoan parasite of endothermic vertebrates including humans. Domestic cats (*Felis catus*) and other members of the family Felidae are the definitive host of *T. gondii* and pass environmentally resistant oocysts in the faeces, which are infective on ingestion to a wide range of intermediate hosts [1]. Following ingestion, oocysts release sporozoites which invade the gut epithelium and transform into tachyzoites, which then multiply rapidly through asexual reproduction and colonize many host tissues, evoking a strong immune response. Tachyzoites differentiate into persistent, slow-growing bradyzoites, a form that produces thick-walled tissue cysts, impervious to the immune response induced by tachyzoites. Bradyzoites are transmitted to either a definitive, or another intermediate host upon ingestion of infected tissues [1].

Despite the existence of a sexual phase in the life cycle, population genetic studies of *T. gondii* in Europe and North America have identified a remarkably clonal population structure, with three predominant clonal lineages, referred to as types I, II and III, accounting for over 84% of strains isolated largely from symptomatic humans and domestic animals [2]. More recently, a fourth lineage (designated haplogroup 12) was identified, principally infecting wild animals in sylvatic niches of North America [3]. The capacity of this parasite to be transmitted asexually by carnivory [4], and the apparent rarity of mixed strain *T. gondii* co-infections among prey species of the definitive felid host (which would promote only self-fertilisation during the sexual cycle), have been interpreted to explain the clonal population genetic structure found in these two regions [5]–[7].

Recent studies of *T. gondii*, especially in wildlife, but also in domestic hosts from parts of the world other than North America and Europe, have reported more genetic diversity [8]–[10]. Although some strains found in these areas appear to be recombinant genotypes derived from crosses between the archetypal clonal lineages, others possess completely novel alleles and are usually referred to as “atypical” or “exotic” strains [11]. It has been suggested that this diversity is driven by regular cycles of sexual reproduction and outcrossing in areas where wildlife plays a more significant role in transmission of the parasite [8], [12], [13]. Even in this situation, however, recent evidence suggests that self-fertilisation is an important step leading to the epidemic expansion and transmission of newly emerged parasite clones [14].

The relative rates of outcrossing and selfing in *T. gondii* are likely to be determined by the frequency of multiple infections (i.e. simultaneous infections with two or more strains) in intermediate hosts, which are subsequently preyed upon by cats. Multiple infections are considered relatively rare events because strong immunity developed in intermediate hosts during primary infections with *T. gondii* is thought to preclude infection with another strain of the parasite [6]. However, concurrent infection with different strains of *T. gondii* is possible, and has been shown to occur in experimental animals [15]. We recently had the opportunity to examine *T. gondii* infections in macropod marsupials (kangaroos, wallaroos and wallabies) sampled from a small, defined geographic area in Western Australia. Our aim was to use direct sequencing of tissue samples to determine the frequency of multiple infections and to examine the genetic diversity of the parasite.

## Materials and Methods

A total of 16 individual adult macropods from three species, *Macropus rufus*, *M. fuliginous* and *M. robustus*, were sampled in 2008 during a routine cull of wild animals by a licensed shooter on a farming property near Menzies, Western Australia (29°41′S, 121°02′E). None of the macropods had any overt clinical symptoms. Tissue samples were collected from five organs (heart, liver, lung, spleen and diaphragm) from each macropod. To avoid any chance of cross-contamination, each organ was first separated from the body and placed into a labelled sterile plastic bag. Multiple 5 g samples were excised, placed into 5 ml sterile tubes for tissue extractions, and samples were analysed at two different laboratories (one at Murdoch University in Australia, one at NIH in the USA) to control for the possibility of spurious amplification during direct PCR on DNA extracted from these tissue samples. All samples were stored at −80°C prior to extraction.

DNA was extracted using the QIAamp Mini Kit (Qiagen; www.qiagen.com), following the manufacturers protocol. Nested PCR was used to amplify the markers *B1*, *SAG2* and *SAG3* [10]. Each 25 µl PCR reaction mixture contained 2.5 µl of 10× PCR buffer, 25 mM MgCl_2_, 0.1 mM deoxynucleoside triphosphate mix (dNTP set-Promega), 5 pmol of each primer, and 0.75 U of TTH plus DNA polymerase. Cycle conditions for the two rounds of PCRs for the *B1* gene were: initial 95°C for 5 min, followed by 30 cycles of 94°C for 30 s, 60°C for 30 s, and 72°C for 90 s. For *SAG2*, the annealing temperatures were 65°C and 63°C for the first and second round respectively, and both rounds ran for 35 cycles. For *SAG3*, the annealing temperature was 60°C and 35 cycles were run for both rounds. The second round PCR used 1 µl of first round reaction, diluted 1∶20, as template. The final PCR product was separated on a 1.5% agarose gel and visualised by staining with i-sybersafe-s33102. Negative PCR controls (i.e. not including template DNA) were included in all reactions, and standard type I and type III template DNA used as positive controls.

Tissue samples which amplified at one or more of the diagnostic *T. gondii* markers were considered positive. Prevalence of parasite infection was expressed as percentage of hosts with a positive DNA sample and 95% confidence intervals were calculated assuming a binomial distribution, using the software Quantitative Parasitology 3.0 [16]. Difference in prevalence among organs was tested by χ^2^.

PCR products from all samples were purified with the Wizsv GEL & PCR or EXoSAP-IT Kits (GE Healthcare Life Sciences) and sequencing reactions performed using a BigDye Terminator v3.1 Cycle Sequencing Kit (Applied Biosystems, Scoresby, Australia). All sequencing was conducted with an Applied Biosystems 3730XL DNA analyser and sequence alignment was conducted using Clustal W [17]. Following alignment, sequences from all samples were compared with type I, II and III reference isolates at the *B1* locus (polymorphisms distinguish type I from type II and III strains, which share the same allele), the *SAG2* locus (polymorphisms distinguish types I and II strains, which share the same allele, from type III) and the *SAG3* locus (polymorphisms distinguish all three strains).

## Results

Among the three loci that were tested, *B1* showed the highest sensitivity and detected *T. gondii* in 45 out of the total of 80 tissue samples. Of these 45 samples, *T. gondii* was detected in 41 and 43 samples at the *SAG2* and *SAG3* loci, respectively. None of the samples that were negative at the *B1* locus tested positive with the other loci. Using the *B1* gene as a diagnostic marker, all 16 individual macropods were infected by *T. gondii*, with the parasite detected in tissue samples from at least one organ (Table 1). Thirteen of the macropods in our study had infections in more than one organ, with all five organs infected in two macropods (Table 1). Over all hosts, there was a significant difference in the prevalence of *T. gondii* in the different organs that were tested (χ^2^ = 9.65, P = 0.04), with the highest infection rate in heart (81.3%, 95% CI 56.4–94.7%), followed by spleen and diaphragm (62.5%, 95% CI 37.2–82.2%), liver (43.8%, 95% CI 20.8–89.4%) and lungs being least heavily infected (31.3% 95% CI 13.2–56.4%).

**Table 1:** Presence of *Toxoplasma gondii* in tissue samples from five different organs in 16 macropod marsupials of three species (*Macropus rufus, M. fuliginosus and M. robustus*) tested by direct PCR.

Animal ID	Species	Organ	Infected?	Animal ID	Species	Organ	Infected?
K-1	*M. rufus*	Heart	positive	K-9	*M. fuliginosus*	Heart	positive
		Liver	negative			Liver	positive
		Lung	positive			Lung	negative
		Spleen	positive			Spleen	positive
		Diaphragm	positive			Diaphragm	positive
K-2	*M. rufus*	Heart	negative	K-10	*M. fuliginosus*	Heart	positive
		Liver	positive			Liver	positive
		Lung	negative			Lung	negative
		Spleen	negative			Spleen	positive
		Diaphragm	positive			Diaphragm	positive
K-3	*M. rufus*	Heart	positive	K-11	*M. fuliginosus*	Heart	positive
		Liver	positive			Liver	positive
		Lung	positive			Lung	positive
		Spleen	positive			Spleen	positive
		Diaphragm	positive			Diaphragm	positive
K-4	*M. rufus*	Heart	positive	K-12	*M. robustus*	Heart	positive
		Liver	negative			Liver	negative
		Lung	negative			Lung	negative
		Spleen	positive			Spleen	positive
		Diaphragm	positive			Diaphragm	negative
K-5	*M. rufus*	Heart	negative	K-13	*M. robustus*	Heart	positive
		Liver	negative			Liver	negative
		Lung	negative			Lung	negative
		Spleen	positive			Spleen	negative
		Diaphragm	negative			Diaphragm	positive
K-6	*M. rufus*	Heart	negative	K-14	*M. robustus*	Heart	positive
		Liver	positive			Liver	negative
		Lung	negative			Lung	positive
		Spleen	negative			Spleen	positive
		Diaphragm	negative			Diaphragm	negative
K-7	*M. fuliginosus*	Heart	positive	K-15	*M. robustus*	Heart	positive
		Liver	negative			Liver	negative
		Lung	negative			Lung	negative
		Spleen	positive			Spleen	negative
		Diaphragm	negative			Diaphragm	negative
K-8	*M. fuliginosus*	Heart	positive	K-16	*M. robustus*	Heart	positive
		Liver	positive			Liver	negative
		Lung	negative			Lung	positive
		Spleen	negative			Spleen	negative
		Diaphragm	positive			Diaphragm	negative

Polymorphisms were detected at 29 single nucleotide positions of the 530 bp *B1* 35 copy tandem gene array. All polymorphisms were point substitutions, rather than insertions or deletions. Of 45 samples that were amplified and sequenced at the *B1* locus, 34 different alleles were detected (Table S1). The sequence data indicated that two samples had an allele identical to the type I reference isolate, two had an allele identical to the type II/III reference isolates, while 41 had non-archetypal alleles, with one or more novel polymorphisms at the *B1* locus (Table S1). In almost all cases, these atypical alleles differed from the type I or type II/III reference alleles by between one and three nucleotide positions (Table S1). Two nucleotide peaks were detected in a number of samples, and these presumably reflected polymorphisms within the 35 copy *B1* gene array. Identical sequences for each unique allele were obtained from independent extractions followed by PCR-DNA sequencing in separate laboratories at Murdoch University and NIH, indicating that it is very unlikely that the novel *B1* alleles were the result of a spurious amplification.

At the 471 bp *SAG2* locus, substitutions were found at six single nucleotide positions. Four different alleles were found among the samples that were amplified at this locus; 16 samples had an allele identical to the type I and II reference isolates, 26 samples had an allele identical to the type III reference isolate, while two samples had atypical alleles, differing at two and three nucleotide positions from the archetypal type III allele (Table S2). Substitutions were found at nine single nucleotide positions at the 225 bp *SAG3* locus, with five different alleles among the samples that were amplified at this locus (Table S2). Seven samples had an allele identical to the type I reference isolate, thirty two samples had an allele identical to the type II reference isolate, 18 had an allele identical to the type III reference isolate, while two had atypical alleles (Table S2). Of these atypical alleles, one differed from the type II reference allele at two nucleotide positions, while the other two differed from the type I reference allele at two nucleotide positions.

Of the 45 different organs from which *T. gondii* could be amplified at either the *SAG2* or *SAG3* locus, 18 (40%) showed mixed nucleotides at one or more positions (Table S2). Three samples had two nucleotide peaks (C and T) at position 40 of the *SAG2* locus, indicating the presence of both type I/II and type III alleles, while one sample had two nucleotide peaks at positions 49 and 76 of the *SAG2* locus, being a putative mixture of type III and an atypical allele. Eighteen samples had two nucleotide peaks at a number of positions of the *SAG3* locus, with seven samples being putative mixtures of types I and II alleles, ten being putative mixtures of types II and III alleles and one being a putative mixture of types I, II and III alleles. Because both *SAG2* and *SAG3* are single-copy genes, these di-nucleotide peaks were likely the result of mixed infections with different genotypes of *T. gondii* in the same organ. All samples with di-nucleotide peaks at either the *SAG2* or *SAG3* locus were independently re-sequenced at Murdoch University and NIH, with the same results being obtained. As a test of our interpretation that di-nucleotide peaks represent mixed infections, marsupial DNA was spiked with limiting concentrations of DNA at a variety of ratios (i.e. 10∶1, 1∶1, 1∶10) from a type I and a type II strain of *T. gondii* and the *SAG2* locus PCR-amplified in five independent reactions. The resulting product was sent for direct DNA sequencing of the PCR population. In all cases, both alleles were amplified, and this was detected by the presence of di-nucleotide peaks at the relevant polymorphic sites between the two alleles (data not shown).

Table 2 shows the multi-locus genotypes at each of the three loci which could be amplified for all samples. All except three samples had non-archetypal alleles for at least one of the three loci that were amplified. Fourteen samples showed evidence of recombination between archetypal genotypes, as they possessed different inheritance patterns of archetypal lineage alleles at two or more loci. Thirteen of the 16 macropods had infections in more than one organ and, of these, all had different multilocus genotypes of *T. gondii* in different organs, indicating multiple infections of individual kangaroos (Table 2). Strict determination of the multi-locus genotypes present in each tissue was confounded by the number of mixed strain multiple infections detected.

**Table 2:** Multi-locus genotypes of *Toxoplasma gondii* by direct PCR and sequencing of tissue samples from macropods.

Sample	Alleles			Genotype	Sample	Alleles			Genotype
	B1	SAG2	SAG3			B1	SAG2	SAG3	
K1-Heart	I	NT	II+III	Recombinant/Mixed	K9-Heart	U-19	III	II	Recombinant
K1-Lung	U-1	NT	II+III	Mixed	K9-Liver	U-20	U-1	II	
K1-Spln	U-2	III	I+II	Recombinant/Mixed	K9-Spln	U-17	I/II	I+II+III	Mixed
K1-Diaph	U-3	I/II	II		K9-Diaph	U-21	III	II+III	Mixed
K2-Liver	U-4	III	II	Recombinant	K10-Heart	U-22	III	III	
K2-Diaph	U-5	III	III		K10-Liver	U-17	III	II+III	Mixed
K3-Heart	U-6	III	III		K10-Spln	U-23	III	III	
K3-Liver	U-7	III	III		K10-Diaph	U-24	III	II	Recombinant
K3-Lung	II/III	III	II+III	Mixed	K11-Heart	I	NT	U-1	
K3-Spln	U-8	I/II	II+III	Mixed	K11-Liver	U-25	II+III	II+III	Mixed
K3-Diaph	U-9	III	II+III	Mixed	K11-Lung	U-26	III	II	Recombinant
K4-Heart	U-7	III	II	Recombinant	K11-Spln	U-27	II+III	II+III	Mixed
K4-Spln	U-11	III	NT		K11-Diaph	U-28	I/II	III	Recombinant
K4-Diaph	U-12	I/II	II		K12-Heart	U-25	I/II	I+II	Mixed
K5-Spln	II/III	III	II	Recombinant	K12-Spln	U-29	I/II	I+II	Mixed
K6-Liver	U-13	III	I+II+III	Mixed	K13-Heart	U-30	III	I+II	Recombinant/Mixed
K7-Heart	U-14	I/II	II		K13-Diaph	U-31	I/II	I+II	Mixed
K7-Spln	U-15	II+III	II+III	Mixed	K14-Heart	U-32	I/II	U-2	
K7-Diaph	U-16	III	II	Recombinant	K14-Lung	U-33	I/II	II	
K8-Heart	U-15	NT	II+III	Mixed	K14-Spln	U-27	III	II	Recombinant
K8-Liver	U-17	III	I+II	Recombinant/Mixed	K15-Heart	U-30	I/II	U-2	
K8-Diaph	U-18	III	I+II	Recombinant/Mixed	K16-Heart	U-28	I/II	II	
					K16-Lung	U-33	I/II	NT	

U indicates non-archetypal allele. I, II and III refer to archetypal alleles from type I, II and III strains. NT indicates that the sample was not amplified.

## Discussion

Studies of *T. gondii* in domestic cycles of transmission in Europe and North America have typically found high rates of seroprevalence (>40%) in people, livestock and companion animals [7]. High prevalences have also been reported in wildlife in different areas of the world, for example approximately 50% in wild boar (*Sus scrofa*) in France [18], 60% in white-tailed deer (*Odocoileus virginianus*) in the USA [19], 75% in capybara (*Hydrochaeris hydrochaeris*) in Brazil [20] and 84% in black bears (*Ursus americanus*) in the USA [21]. There have, however, been few studies of the prevalence of *T. gondii* in either domestic or wildlife cycles in Australia. In this study, we found that of 16 macropod marsupials of three different species inhabiting a defined geographic area in the arid rangelands of Western Australia, all were infected. This is much greater than the prevalence found in three serological studies on macropods in different areas of Australia; 8.5% among *Macropus rufogriseus* and *Thylogale billardierii* in Tasmania [22], 15.5% and among *Macropus fuliginosus* in south western Australia [23] and 5% among *Petrogale penicillata* in southeast Queensland [24]. This suggests the need for serological studies on a larger sample of macropods from our study site, to determine whether our results are really reflective of a higher prevalence of *T. gondii* in this region.

It has been suggested that Australian native mammals are particularly prone to severe pathological effects when infected with *T. gondii*, possibly from not being exposed to the parasite prior to the introduction of domestic cats some 300 years ago [25]. In our study, there were no clinical signs of toxoplasmosis in the wild macropods sampled, despite a high prevalence of infection, often in a number of different organs. This does not necessarily mean, however, that the high infection rates found in these animals has no implications for wildlife conservation in Australia. Virulence is not a static property of a parasite's genome and strains of *T. gondii* that are avirulent in one host species may be virulent in another [10]. In addition, most clinical cases of toxoplasmosis in Australian wildlife have been in captive animals, suggesting that non-parasite factors, such as immune or nutritional status of the host, also affect virulence [10].

Macropods are herbivores, so presumably the source of infection in this study was oocysts shed by definitive felid hosts, probably feral cats (*Felis catus*), although vertical transmission has also been reported in kangaroos [26] and may play a role in maintaining the life cycle of *T. gondii* in this arid rangeland region. The high prevalence of infection suggests heavy environmental contamination of this area with oocysts. Feral cats are known to be present in the area, and, although population size is not known, studies in similar arid environments in Australia have estimated cat densities at between 0.75 and 2.8 km^−2^ [27], [28]. Recent studies in our laboratory (data not shown) have confirmed *T. gondii* infection in four feral cats sampled from the south west of Western Australia, although we have no data on *T.gondii* infections in feral cats in the area from which the macropods were sampled in this study or from other areas of the State.

Our sampling design, involving direct detection and sequencing of DNA from tissue samples, imposed a high demand for PCR sensitivity and therefore limited the extent of multilocus genotyping that could be performed. We were able to obtain sequencing data from only three loci; *B1*, *SAG2* and *SAG3*. Nevertheless, the allelic diversity at these three loci suggested that most genotypes exist as minor variants of established archetypal lineages by the accumulation of new mutations, particularly at the *B1* locus, that had subtly altered their genetic fingerprint. However, it was also clear that for 14 “variants” recombination between archetypal lineages followed by genetic drift was responsible for the diversification of these strains. Specifically, atypical alleles (i.e. different to archetypal type I, II or III strains) were found in 41 samples sequenced at the *B1* locus, one sample at the *SAG2* locus and three samples at the *SAG3* locus. Parameswaran et al. [10] also found a large number of atypical alleles in *T. gondii* infecting Australian wildlife, which they interpreted as a consequence of genetic drift following the isolation of archetypal strains imported into Australia during early European settlement. As in the present study, Parameswaran et al. [10] also found evidence of recombination between these archetypal strains. These results suggest that genetic diversity may be enhanced by regular cycles of outcrossing between different strains of *T. gondii* infecting feral cats, as has been suggested for felid definitive hosts involved in wildlife transmission cycles in other areas of the world [8]. We should stress, however, that our results are only suggestive; confirmation of the extent and nature of genetic diversity in *T. gondii* infecting Australian wildlife will require isolation, cloning and further sequencing studies using a wider range of loci.

The most important finding from our study was that 14 of the 16 infected macropods harboured more than one genotype of *T. gondii*, as indicated by the presence of dinucleotide peaks from sequencing the single locus *SAG2* and *SAG3* genes in samples from one organ or by the presence of different multilocus genotypes in different organs of the same host. Although multiple infections have previously been reported in naturally infected intermediate hosts [20], [29], they are often considered to be relatively rare events because of the strong immunity developed by intermediate hosts to primary infections with *T. gondii* [6]. The unique aspect of our study is the extremely high rate of multiple infections in intermediate hosts sampled from a defined geographic area. These results may suggest something unusual about macropod marsupials as hosts or the transmission cycle operating in this area of arid Australia, or it may be a consequence of our sampling design, which employed direct extraction and sequencing of *T. gondii* DNA from a number of different organs from each host.

Direct detection of DNA, while it limits the number of loci which can be amplified and examined, may be more likely to detect multiple infections than traditional techniques of parasite isolation, because of the potential for sampling bias arising from competition between different strains when grown *in vitro* or *in vivo* [7], [13]. In addition, it is rare for studies of *T. gondii* in intermediate hosts to sample a range of different organs. Of 13 macropods which had *T. gondii* infections in more than one organ, all had different genotypes of the parasite in these organs. There was also some evidence of multiple infections in the same organ, but that occurred in only 44% of samples. These results suggest that examining only one organ for *T. gondii* may lead to an underestimation of multiple infections. One of the disadvantages of direct detection of DNA is the possibility of DNA contamination producing spurious results. While this can never be ruled out completely, we took a number of precautions to avoid contamination. These included adding negative controls to every PCR run and repeating DNA extractions from a number of tissue samples, with the same genotypes being consistently detected. These precautions, plus the level of genetic diversity found and replicated in two different laboratories, give us confidence in the robustness of our results.

The significance of multiple infections of *T. gondii* in intermediate hosts is the potential it provides for genetic exchange during the sexual phase of the parasites life cycle. It has previously been assumed that, because of the transient nature of infections in the definitive host, and the apparent rarity of identifying multiple infections in intermediate hosts, most infections within cats involve only a single genotype of *T. gondii* derived from a single source of prey [5], [6], [7]. This would lead to effective self fertilisation during sexual reproduction and the perpetuation of a clonal population structure. On the other hand, if intermediate hosts are usually infected with a number of different genotypes of *T. gondii*, cross fertilisation is more likely to occur during sexual reproduction, which would break up clonal genotypes and lead to a more panmictic population structure.

The results from our study suggest that, at least in sylvatic cycles of transmission in Australia, multiple infections of intermediate hosts may be very common. While the species of macropod marsupials we sampled are too large to be preyed upon by feral cats, the diversity of genotypes they contain is likely to be indicative of a similar diversity in oocysts shed by cats. We hypothesise that further studies of *T. gondii* in both feral cats and small mammals preyed upon by cats in the area will find a large proportion of multiple infections with different genotypes.

## Supporting Information

Table S1 **Polymorphisms in the** ***B1*** **gene of** ***Toxoplasma gondii*** **by direct PCR and sequencing of tissue samples from macropods.** Nucleotide positions refer to sites in published GenBank sequences. “.” indicates identity with type I reference sequence. U indicates non-archetypal allele. I and II/III refer to archetypal alleles from Type I and Type II or III strains.
(DOC)

Table S2 **Polymorphisms in the** ***SAG2*** **and** ***SAG3*** **genes of** ***Toxoplasma gondii*** **by direct PCR and sequencing of tissue samples from macropods.** Nucleotide positions refer to sites in published GenBank sequences. “.” indicates identity with type I reference sequence. U indicates non-archetypal allele. I, II and III refer to archetypal alleles from type I, II and III strains. NT indicates that the sample was not amplified.
(DOC)
