# Supplementary material for: Western Australian Marsupials Are Multiply Infected with Genetically Diverse Strains of Toxoplasma gondii
Source: PLoS One. 2012 Sep 24;7(9):e45147. doi: 10.1371/journal.pone.0045147 (PMC3454407; doi:10.1371/journal.pone.0045147)
Supplement: Table S1 — Polymorphisms in the B1 gene of Toxoplasma gondii by direct PCR and sequencing of tissue samples from macropods. Nucleotide positions refer to sites in published GenBank sequences. “.” indicates identity with type I reference sequence. U indicates non-archetypal allele. I and II/III refer to archetypal alleles from Type I and Type II or III strains. (DOC) [file pone.0045147.s001.doc]

**Table S1.** Polymorphisms in the *B1* gene of *Toxoplasma gondii* by direct PCR and sequencing of tissue samples from macropods. Nucleotide positions refer to sites in published GenBank sequences. “.” indicates identity with type I reference sequence. U indicates non-archetypal allele. I and II/III refer to archetypal alleles from Type I and Type II or III strains.

| Sample | Nucleotide | | | | | | | | | | | | | | | | | | | | | | | | | | | | | Allele |
| --- | --- | --- | --- | --- | --- | --- | --- | --- | --- | --- | --- | --- | --- | --- | --- | --- | --- | --- | --- | --- | --- | --- | --- | --- | --- | --- | --- | --- | --- | --- |
|  | 185 | 209 | 228 | 252 | 257 | 258 | 275 | 284 | 312 | 326 | 334 | 338 | 343 | 366 | 404 | 411 | 418 | 470 | 504 | 510 | 514 | 525 | 532 | 533 | 552 | 559 | 597 | 611 | 646 |  |
| Type I | T | C | T | A | A | G | G | A | G | C | A | A | A | T | A | A | T | A | G | A | A | A | T | A | C | G | T | A | T | I |
| Type II/III | . | . | . | . | . | . | . | . | . | . | . | . | . | C/T | . | . | . | . | G/C | . | . | . | . | . | . | . | . | . | . | II/III |
| K1-Heart | . | . | . | . | . | . | . | . | . | . | . | . | . | . | . | . | . | . | . | . | . | . | . | . | . | . | . | . | . | I |
| K1-Lung | . | . | . | . | . | . | . | . | . | . | . | . | . | C | . | . | . | . | . | . | . | . | . | C | . | A | . | . | . | U-1 |
| K1-Spln | . | . | . | . | . | T | . | G | . | . | . | . | . | C | . | . | . | . | . | . | . | . | . | C | . | . | . | . | . | U-2 |
| K1-Diaph | . | . | . | . | . | . | . | . | . | . | . | . | . | C | . | . | . | . | G/C | . | . | . | . | . | . | . | . | A/G | . | U-3 |
| K2-Liver | . | . | . | . | . | . | . | . | . | . | . | . | . | . | . | . | . | . | C | . | . | . | . | . | . | . | . | . | . | U-4 |
| K2-Diaph | . | . | . | A/G | . | . | . | . | . | . | . | A/G | . | . | . | . | C/T | . | . | . | . | . | . | . | . | . | . | . | . | U-5 |
| K3-Heart | . | . | . | . | . | . | . | . | . | . | . | . | . | . | . | . | . | . | . | . | . | . | . | A/C | . | . | . | . | . | U-6 |
| K3-Liver | . | . | . | . | . | . | . | . | . | . | . | . | . | C/T | . | . | . | . | . | . | . | . | . | A/C | . | . | . | . | . | U-7 |
| K3-Lung | . | . | . | . | . | . | . | . | . | . | . | . | . | C/T | . | . | . | . | G/C | . | . | . | . | . | . | . | . | . | . | II/III |
| K3-Spln | . | . | . | . | A/G | . | . | . | . | . | . | . | . | C | A/G | . | . | . | . | . | . | . | . | C | . | . | . | . | . | U-8 |
| K3-Diaph | . | . | . | . | . | . | . | . | . | . | . | . | G | . | . | . | . | . | . | . | . | . | . | . | G | . | . | . | . | U-9 |
| K4-Heart | . | . | . | . | . | . | . | . | . | . | . | . | . | C/T | . | . | . | . | . | . | . | . | . | A/C | . | . | . | . | . | U-7 |
| K4-Spln | . | T | . | . | . | . | . | . | . | . | . | . | . | . | . | . | . | . | . | . | . | . | . | . | . | . | . | . | . | U-10 |
| K4-Diaph | . | . | . | . | . | . | . | . | . | . | . | . | . | . | . | . | . | . | . | . | . | . | . | . | . | . | . | . | C | U-11 |
| K5-Spln | . | . | . | . | . | . | . | . | . | . | . | . | . | C/T | . | . | . | . | G/C | . | . | . | . | . | . | . | . | . | . | II/III |
| K6-Liver | . | . | . | . | . | . | . | . | . | . | . | . | . | . | . | . | . | . | G/C | . | . | . | T/C | . | . | . | . | . | . | U-12 |
| K7-Heart | . | . | . | . | . | . | . | . | . | T | . | . | . | C | . | A/G | . | . | . | . | . | . | . | C | . | . | . | . | . | U-13 |
| K7-Spln | . | . | C/T | . | . | . | . | . | . | . | . | . | . | C/T | . | . | . | . | G/C | . | . | . | . | A/C | . | . | . | A/G | . | U-14 |
| K7-Diaph | . | . | . | . | . | . | . | . | . | . | . | . | . | C | . | G | . | . | C | . | . | . | . | . | . | . | . | . | . | U-15 |
| K8-Heart | . | . | C/T | . | . | . | . | . | . | . | . | . | . | C/T | . | . | . | . | G/C | . | . | . | . | . | . | . | . | . | . | U-16 |
| K8-Liver | . | . | . | . | . | . | . | . | . | . | . | . | . | C/T | . | . | . | . | . | . | . | . | . | . | . | . | . | . | . | U-17 |
| K8-Diaph | . | . | . | . | . | . | . | . | G/C | . | . | A/G | . | . | . | . | C/T | A/G | . | . | . | A/G | . | . | . | . | . | . | . | U-18 |
| K9-Heart | . | . | . | . | . | . | . | . | . | . | . | . | . | . | . | . | . | . | G/C | . | . | . | . | . | . | . | . | . | . | U-19 |
| K9-Liver | . | . | . | . | . | . | . | . | . | . | A/G | A/G | . | . | . | . | C/T | . | . | . | . | A/G | . | . | . | . | . | . | . | U-20 |
| K9-Spln | . | . | . | . | . | . | . | . | . | . | . | . | . | C/T | . | . | . | . | . | . | . | . | . | . | . | . | . | . | . | U-17 |
| K9-Diaph | . | . | . | . | . | . | . | A/G | . | . | . | . | . | . | . | . | . | . | G/C | . | . | . | . | . | . | . | . | . | . | U-21 |
| K10-Heart | . | . | . | . | . | . | A/G | . | . | . | . | . | . | C/T | . | . | . | . | . | . | . | . | . | . | . | . | . | . | . | U-22 |
| K10-Liver | . | . | . | . | . | . | . | . | . | . | . | . | . | C/T | . | . | . | . | . | . | . | . | . | . | . | . | . | . | . | U-17 |
| K10-Spln | A/T | . | . | . | . | . | . | . | . | . | . | . | . | . | . | . | . | . | . | . | . | . | . | . | . | . | . | . | . | U-23 |
| K10-Diaph | . | . | . | . | . | . | . | . | . | . | . | A/G | . | . | . | . | . | . | . | A/G | . | . | . | . | G | . | . | . | . | U-24 |
| K11-Heart | . | . | . | . | . | . | . | . | . | . | . | . | . | . | . | . | . | . | . | . | . | . | . | . | . | . | . | . | . | I |
| K11-Liver | . | . | . | . | . | . | . | . | . | . | . | . | . | C | . | . | . | . | . | . | . | . | . | . | . | . | . | . | . | U-25 |
| K11-Lung | . | . | . | . | . | . | . | A/G | . | . | . | . | . | C | . | . | . | . | . | . | . | . | . | C | . | . | . | . | . | U-26 |
| K11-Spln | . | . | . | . | . | . | . | . | . | . | . | . | . | C | . | . | . | . | . | . | . | . | . | C | . | . | . | . | . | U-27 |
| K11-Diaph | . | . | . | . | . | . | . | . | . | . | . | . | . | C | . | . | . | . | C | . | . | . | . | . | . | . | . | G | . | U-28 |
| K12-Heart | . | . | . | . | . | . | . | . | . | . | . | . | . | C | . | . | . | . | . | . | . | . | . | . | . | . | . | . | . | U-25 |
| K12-Spln | . | . | . | . | . | . | . | . | . | . | . | . | . | C | . | . | . | . | G/C | . | . | . | . | A/C | . | . | . | . | . | U-29 |
| K13-Heart | . | . | . | . | . | . | . | . | . | . | . | . | . | C/T | . | . | . | . | G/C | . | . | . | . | A/C | . | . | C/T | A/G | . | U-30 |
| K13-Diaph | . | . | C/T | . | . | . | . | . | . | . | . | . | . | C/T | . | . | . | . | G/C | . | . | . | . | A/C | . | . | . | A/G | . | U-14 |
| K14-Heart | . | . | . | . | . | . | . | . | . | . | . | . | . | C | . | . | . | . | G/C | . | . | . | . | . | . | . | . | . | . | U-31 |
| K14-Lung | . | . | . | . | . | . | . | . | . | . | . | . | . | C | . | . | . | . | . | . | . | . | . | A/C | . | . | . | . | . | U-32 |
| K14-Spln | . | . | . | . | . | . | . | . | . | . | . | . | . | C | . | . | . | . | . | . | . | . | . | C | . | . | . | . | . | U-27 |
| K15-Heart | . | . | . | . | . | . | . | . | . | . | . | . | . | C/T | . | . | . | . | G/C | . | . | . | . | A/C | . | . | C/T | A/G | . | U-30 |
| K16-Heart | . | . | . | . | . | . | . | . | . | . | . | . | . | C | . | . | . | . | C | . | . | . | . | . | . | . | . | . | . | U-33 |
| K16-Lung | . | . | . | . | . | . | . | . | . | . | . | . | . | C | . | . | . | . | . | . | . | . | . | A/C | . | . | . | . | . | U-32 |
