# Supplementary material for: Western Australian Marsupials Are Multiply Infected with Genetically Diverse Strains of Toxoplasma gondii
Source: PLoS One. 2012 Sep 24;7(9):e45147. doi: 10.1371/journal.pone.0045147 (PMC3454407; doi:10.1371/journal.pone.0045147)
Supplement: Table S2 — Polymorphisms in the SAG2 and SAG3 genes of Toxoplasma gondii by direct PCR and sequencing of tissue samples from macropods. Nucleotide positions refer to sites in published GenBank sequences. “.” indicates identity with type I reference sequence. U indicates non-archetypal allele. I, II and III refer to archetypal alleles from type I, II and III strains. NT indicates that the sample was not amplified. (DOC) [file pone.0045147.s002.doc]

**Table S2.** Polymorphisms in the *SAG2* and *SAG3* genes of *Toxoplasma gondii* by direct PCR and sequencing of tissue samples from macropods. Nucleotide positions refer to sites in published GenBank sequences. “.” indicates identity with type I reference sequence. U indicates non-archetypal allele. I, II and III refer to archetypal alleles from type I, II and III strains. NT indicates that the sample was not amplified.

| Sample | *SAG2* | | | | | | | *SAG3* | | | | | | | | |  |
| --- | --- | --- | --- | --- | --- | --- | --- | --- | --- | --- | --- | --- | --- | --- | --- | --- | --- |
|  | Nucleotide | | | | | | Allele | Nucleotide | | | | | | | | | Allele |
|  | 32 | 40 | 49 | 52 | 76 | 171 |  | 981 | 1001 | 1005 | 1037 | 1044 | 1046 | 1053 | 1061 | 1076 |  |
| Type I | C | T | T | T | T | T | I | C | G | A | T | G | G | A | G | A | I |
| Type II | . | . | . | . | . | . | II | T | A | G | . | A | . | C | C | G | II |
| Type III | . | C | . | . | . | . | III | . | . | . | C | . | A | C | C | . | III |
| K1-Heart | NT | NT | NT | NT | NT | NT |  | C/T | A/G | A/G | C/T | A/G | A/G | C | C | A/G | II+III |
| K1-Lung | NT | NT | NT | NT | NT | NT |  | . | . | c | C | . | A | C | C | . | III |
| K1-Spln | . | C | . | . | . | . | III | NT | NT | NT | NT | NT | NT | NT | NT | NT |  |
| K1-Diaph | . | . | . | . | . | . | I/II | T | A | G | . | A | . | C | C | G | II |
| K2-Liver | . | C | . | . | . | . | III | T | A | G | . | A | . | C | C | G | II |
| K2-Diaph | . | C | . | . | . | . | III | C/T | A/G | A/G | C/T | A/G | A/G | C | C | A/G | II+III |
| K3-Heart | . | C | . | . | . | . | III | . | . | . | C | . | A | C | C | . | III |
| K3-Liver | . | C | . | . | . | . | III | . | . | . | C | . | A | C | C | . | III |
| K3-Lung | . | C | . | . | . | . | III | . | . | . | C | . | A | C | C | . | III |
| K3-Spln | . | . | . | . | . | . | I/II | C/T | A/G | A/G | C/T | A/G | A/G | C | C | A/G | II+III |
| K3-Diaph | . | C | C/T | . | C/T | . | III+U1 | C/T | A/G | A/G | C/T | A/G | A/G | C | C | A/G | II+III |
| K4-Heart | . | C | . | . | . | . | III | T | A | G | . | A | . | C | C | G | II |
| K4-Spln | . | C | . | . | . | . | III | NT | NT | NT | NT | NT | NT | NT | NT | NT |  |
| K4-Diaph | . | . | C | . | C | . | U1 | T | A | G | . | A | . | C | C | G | II |
| K5-Spln | . | C | . | . | . | . | III | T | A | G | . | A | . | C | C | G | II |
| K6-Liver | . | C | . | . | . | . | III | C/T | A/G | A/G | . | A/G | . | A/C | C/G | A/G | I+II |
| K7-Heart | . | . | . | . | . | . | I/II | T | A | G | . | A | . | C | C | G | II |
| K7-Spln | . | C/T | . | . | . | . | I/II+III | C/T | A/G | A/G | C/T | A/G | A/G | C | C | A/G | II+III |
| K7-Diaph | . | C | . | . | . | . | III | T | A | G | . | A | . | C | C | G | II |
| K8-Heart | NT | NT | NT | NT | NT | NT |  | C/T | A/G | A/G | C/T | A/G | A/G | C | C | A/G | II+III |
| K8-Liver | . | C | . | . | . | . | III | C/T | A/G | A/G | . | A/G | . | A/C | C/G | A/G | I+II |
| K8-Diaph | . | C | . | . | . | . | III | C/T | A/G | A/G | . | A/G | . | A/C | C/G | A/G | I+II |
| K9-Heart | . | C | . | . | . | . | III | T | A | G | . | A | . | C | C | G | II |
| K9-Liver | T | C | . | C | . | C | U-2 | T | A | G | . | A | . | C | C | G | II |
| K9-Spln | . | . | . | . | . | . | I/II | C | A/G | . | C/T | . | A/G | C | C | . | I+II+III |
| K9-Diaph | . | C | . | . | . | . | III | C/T | A/G | A/G | C/T | A/G | A/G | C | C | A/G | II+III |
| K10-Heart | . | C | . | . | . | . | III | C | . | . | C | . | A | C | C | . | III |
| K10-Liver | . | C | . | . | . | . | III | C/T | A/G | A/G | C/T | A/G | A/G | C | C | A/G | II+III |
| K10-Spln | . | C | . | . | . | . | III | T | A | G | . | A | . | C | C | G | III |
| K10-Diaph | . | C | . | . | . | . | III | T | A | G | . | A | . | C | C | G | II |
| K11-Heart | NT | NT | NT | NT | NT | NT |  | T | . | . | . | A | . | C | C | G | U-1 |
| K11-Liver | . | C/T | . | . | . | . | I/II+III | C/T | A/G | A/G | C/T | A/G | A/G | C | C | A/G | II+III |
| K11-Lung | . | C | . | . | . | . | III | T | A | G | . | A | . | C | C | G | II |
| K11-Spln | . | C/T | . | . | . | . | I/II+III | C/T | A/G | A/G | C/T | A/G | A/G | C | C | A/G | II+III |
| K11-Diaph | . | . | . | . | . | . | I/II | . | . | . | C | . | A | C | C | . | III |
| K12-Heart | . | . | . | . | . | . | I/II | C/T | A/G | A/G | . | A/G | . | A/C | C/G | A/G | I+II |
| K12-Spln | . | . | . | . | . | . | I/II | C/T | A/G | A/G | . | A/G | . | A/C | C/G | A/G | I+II |
| K13-Heart | . | C | . | . | . | . | III | C/T | A/G | A/G | . | A/G | . | A/C | C/G | A/G | I+II |
| K13-Diaph | . | . | . | . | . | . | I/II | C/T | A/G | A/G | . | A/G | . | A/C | C/G | A/G | I+II |
| K14-Heart | . | . | . | . | . | . | I/II | . | . | . | . | . | . | C | C | . | U-2 |
| K14-Lung | . | . | . | . | . | . | I/II | T | A | G | . | A | . | C | C | G | II |
| K14-Spln | . | C | . | . | . | . | III | T | A | G | . | A | . | C | C | G | II |
| K15-Heart | . | . | . | . | . | . | I/II | . | . | . | . | . | . | C | C | . | U-2 |
| K16-Heart | . | . | . | . | . | . | I/II | T | A | G | . | A | . | C | C | G | II |
| K16-Lung | . | . | . | . | . | . | I/II | NT | NT | NT | NT | NT | NT | NT | NT | NT |  |
